# Supplementary material for: The Construction and Exploration of a Comprehensive MicroRNA Centered Regulatory Network in Foxtail Millet (Setaria italica L.)
Source: Front Plant Sci. 2022 May 6;13:848474. doi: 10.3389/fpls.2022.848474 (PMC9121102; doi:10.3389/fpls.2022.848474)
Supplement: Supplementary file 3 [file Data_Sheet_3.zip › Sit-miR2118b_Seita.6G156100.1_1004_TPlot.pdf]

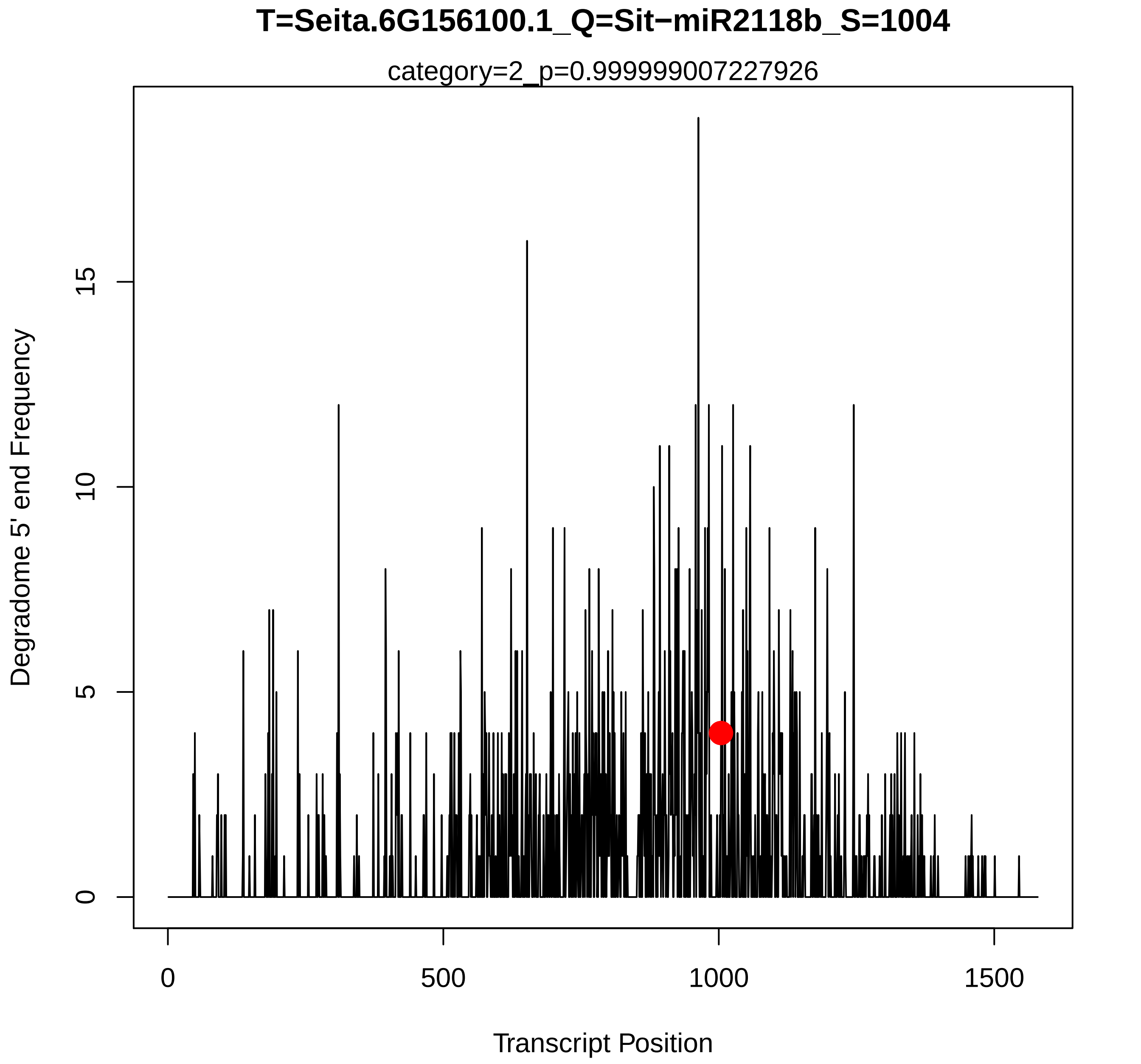
T=Seita.6G156100.1_Q=Sit−miR2118b_S=1004
category=2_p=0.999999007227926
Degradome 5' end Frequency
0
5
10
15
0
500
1000
1500
Transcript Position
